# Biomechanics of common fixation devices for first tarsometatarsal joint fusion—a comparative study with synthetic bones

**DOI:** 10.1186/s13018-018-0876-0

**Published:** 2018-07-11

**Authors:** Rene Burchard, Robin Massa, Christian Soost, Wolfgang Richter, Gerhard Dietrich, Arne Ohrndorf, Hans-Jürgen Christ, Claus-Peter Fritzen, Jan Adriaan Graw, Jan Schmitt

**Affiliations:** 1 Department of Health, University of Witten/Herdecke, Witten, Germany; 2 Department of Trauma and Orthopaedic Surgery, Kreisklinikum Siegen, Weidenauer Str. 76, 57076 Siegen, Germany; 3 School of Science and Technology, University of Siegen, Siegen, Germany; 4 Department of Statistics an Econometrics, University of Siegen, Kohlbettstr, 15, 57072 Siegen, Germany; 5 Department of Mechanical Engineering, University of Siegen, Paul-Bonatz-Str. 9-11, 57076 Siegen, Germany; 6 Department of Anesthesiology and Operative Intensive Care Medicine, Charité—Universitätsmedizin Berlin, Campus Virchow-Klinikum, Augustenburger Platz 1, 13353 Berlin, Germany; 7 Berlin Institute of Health, Berlin, Germany; 8 Department of Orthopaedics and Trauma Surgery, Lahn-Dill-Kliniken Wetzlar, Forsthausstraße 1, 35578 Wetzlar, Germany

**Keywords:** Lapidus fusion, Angle stable, Locking plate, Intramedullary fixation device, Hallux valgus

## Abstract

**Background:**

Hallux valgus disease is a common deformity of the forefoot. There are currently more than 100 surgical approaches for operative treatment. Because hypermobility of the first tarsometatarsal joint is considered to be causal for hallux valgus disease, fusion of the tarsometatarsal joint is an upcoming surgical procedure. Despite the development of new and increasingly stable fixation devices like different locking plates, malunion rates have been reported in 5 to 15% of cases.

**Methods:**

Biomechanical comparison of three commonly used fixation devices (a dorsal locking plate, a plantar locking plate, and an intramedullary fixation device) was performed by weight-bearing simulation tests on synthetic bones. Initial compression force and stiffness during simulation of postoperative weight-bearing were analysed.

**Results:**

Fixation of the first tarsometatarsal joint with the plantar plate combination demonstrated a higher stiffness compared to fixation with the intramedullary implant or the medial locking plate. The intramedullary device provided the highest initial compression force. Failure was detected in the following ranking: (1) the angle-stable intramedullary fixation device, (2) the medial located plate, and (3) the plantar locking plate.

**Conclusion:**

The intramedullary device demonstrated the highest initial compression force of the three tested implants. The plantar locking plate showed the best overall stability during weight-bearing simulation. Further clinical research is necessary to analyse if the intramedullary fixation device needs a longer period of non-weight-bearing to reach a better non-union rate compared to the plantar locking plate.

## Background

Hallux valgus disease is a very common deformity of the forefoot and was first described by Carl Hueter in 1870 [1, 2]. It is defined by a lateral deviation of the great toe and a medial deviation of the first metatarsal bone [1].

While non-operative treatment only reduces symptoms like pain and inflammation but cannot improve the deformity of the bone, operative treatment is much more common. However, there are more than 100 surgical approaches for treatment of hallux valgus disease. There have been attempts to develop treatment algorithms based on the degrees of deviation of the first metatarsal bone and including co-variables such as joint degeneration or incongruity [3, 4].

Hypermobility of the first tarsometatarsal joint is considered to be causal for hallux valgus disease [1]. Therefore, fusion of the first tarsometatarsal joint is an upcoming surgical procedure in the treatment of hallux valgus deformity [5]. In the past, the so-called Lapidus procedure, as a combined fusion of the first tarsometatarsal joint and the medial and intermediate cuneiform bones, often performed as a classical fixation with two crossed screws, has been described as a standard technique by many authors [6, 7]. Based on the Lapidus procedure, modified techniques with an isolated fusion of the first tarsometatarsal joint were developed [8–10]. Progressive developments of new fixation devices such as newly designed locking plates make early weight-bearing and a more comfortable postoperative treatment possible [11]. However, malunion rates between 5 and 15% have been reported [12]. Therefore, one might assume that locking implants will replace the screw procedures to avoid longer postoperative immobilisation periods and lower malunion rates [11]. In addition, high initial compression forces between bone fragments are a predictor for a good consolidation between the fragments in procedures such as osteosyntheses, osteotomies, and arthrodeses [13]. New developments in hardware design and implant techniques combined with the patients’ expectations for a fast recovery and return to normal life have triggered recent research for the best implant device and surgical procedure. Many research groups have compared various hardware solutions such as crossed screws, dorsal and plantar locking plates, and intramedullary locking devices in cadaver or synthetic bone studies [12, 14–16]. Nevertheless, the actual literature shows heterogeneous results referring to recent fixation concepts in this field of foot surgery.

However, all surgical procedures to fix the tarsometatarsal joint in hallux valgus disease should provide a minimal non-union rate with a maximum of postoperative comfort and safety for the patient. Therefore, the aim of this study was to investigate, if common and innovative surgical approaches using a medial locking plate, a plantar locking plate, or an intramedullary locking device differ in their initial capacity of compression of the osteosynthesis. In addition, to detect a postinterventional loss of stability including a bone-implant-combination failure, weight-bearing in a healing shoe was simulated and stability of the osteosynthesis was compared among groups.

## Methods

### Experimental set-up

Nine (*n* = 9) composite synthetic bone pairs (First Metatarsal Model 3423 and Cuneiform Model 3421-1, Sawbones Europe AB, Malmö, Sweden) were analysed. Synthetic bones are considered to have similar structural and mechanical properties like natural bones. A potential bias associated with different bone shapes, bone qualities, or bone sizes when using cadaveric specimens is eliminated by using synthetic bone [17, 18].

**Fig. 1:**
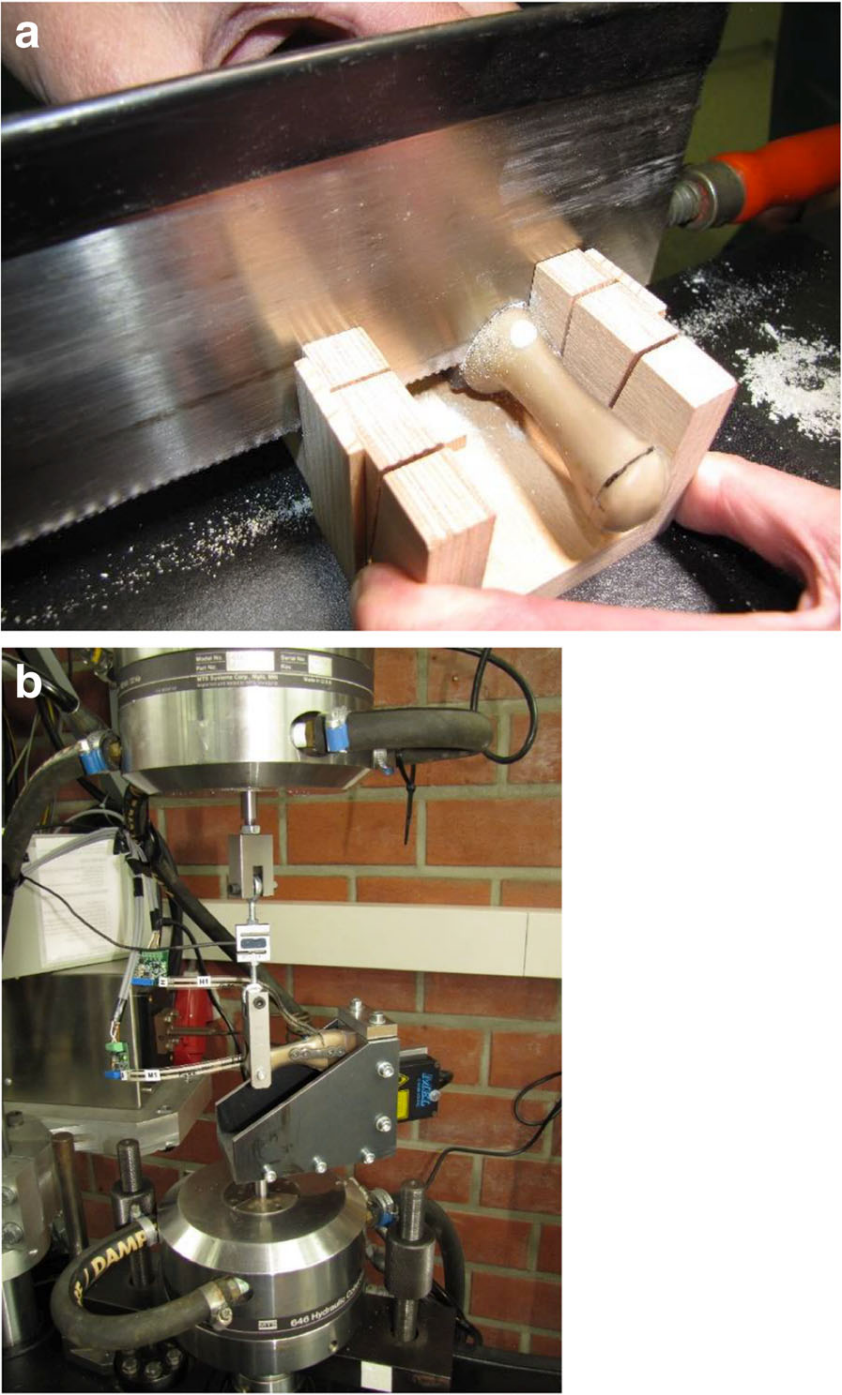
**a** Sawing device to perform completely reproducible cuts for the fusion sides. **b** Mounting device and experimental set-up. The model was fixed in the device at the bottom. Force was applied from the top as a tensile force measured by a sensor. Displacement of the bones was measured b < a laser in the mounting device

A typical arthrodesis of the first tarsometatarsal joint with a tangential cut of articular sides was performed, and a pressure sensor (FlexiForce® Load/Force Sensor, Tekscan, South Boston, USA) was inserted into the fusion gap between the first metatarsal and the cuneiform synthetic bone before fixation of the implant. A special sawing template was used to guarantee that all osteotomies were performed with a repetitious accuracy (Fig. 1a). Three bone-implant-combinations were tested, and experiments were run in triplicates. The same surgeon, certified by the German Society for Foot Surgery, performed each osteosynthesis. After arthrodesis, the cuneiform bone was fixed rigidly in a specially designed mounting device, which was constructed to guide a cyclic sinusoidal testing simulation. Tests were performed in the versatile material testing system MTS 810® (MTS Systems GmbH, Berlin, Germany) with tensile loads to simulate the stress due to the walking process. The forces are measured with an additional load cell (ME KD24S, ME-Meßsysteme GmbH, Hennigsdorf, Germany). A laser (M5L/10®, MEL Mikroelektronik GmbH, Eching, Germany), attached at the mounting device with the fixed cuneiform bone, measured the motion between the cuneiform and the first metatarsal bone (Fig. 1b). Data were recorded by the digital controller MTS Star II® (MTS Systems GmbH, Berlin, Germany) and a HBM Spider8 data acquisition system (HBM GmbH, Darmstadt, Germany).

### Implants

**Fig. 2:**
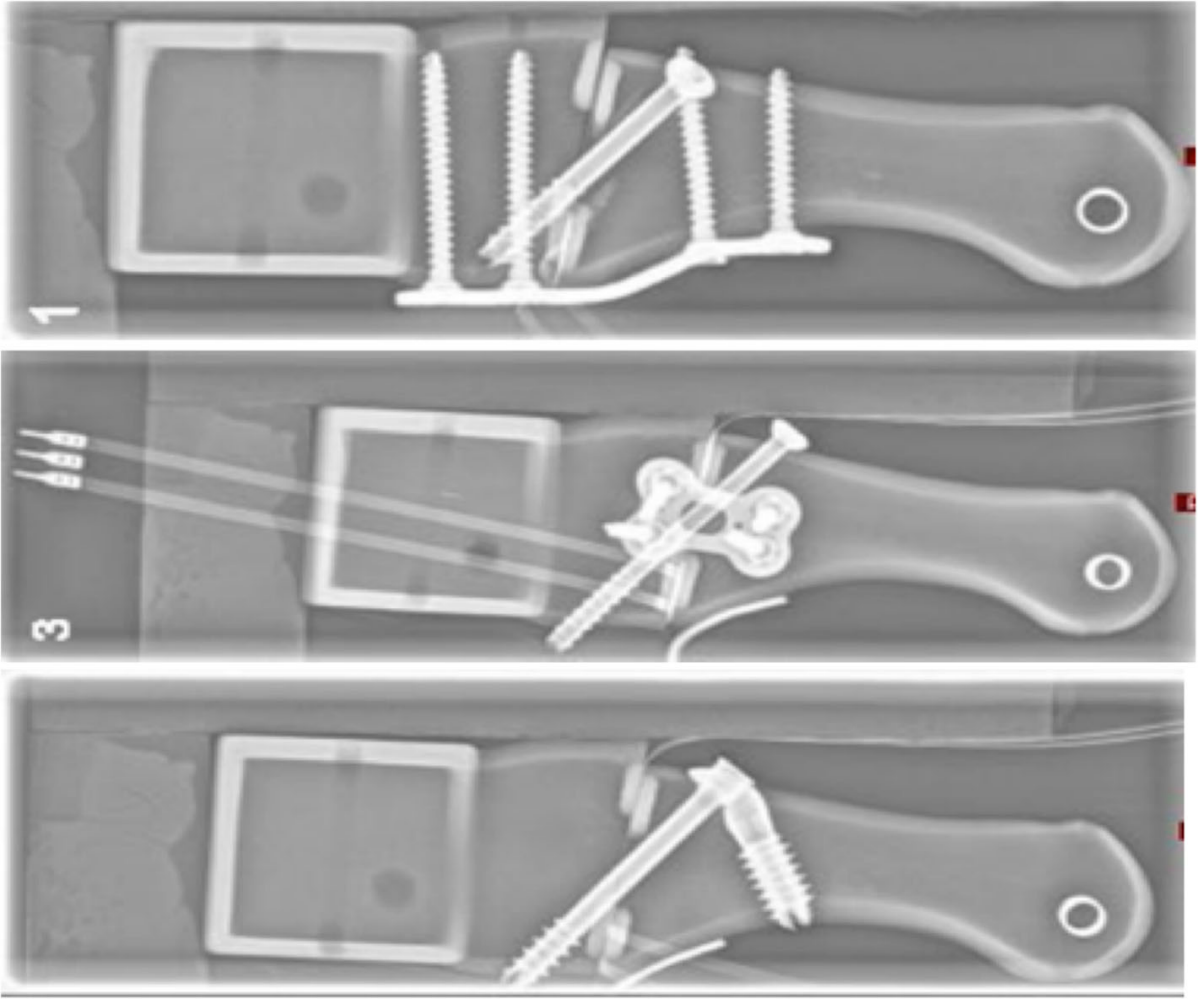
X-ray images of the three models. **a** Plantar locking plat with crossed screw, **b** medial locking plate with crossed screw, and **c** the intramedullary angle-stable implant

Two different extramedullary locking plates and one intramedullary locking fixation device were compared with each other:

*PEDUS L Plantar Lapidus Plate® (Axomed GmbH, Freiburg, Germany)*: a titanium plantar locking plate with four holes for 2.7-mm angle-stable cortical screws, 39 mm long. Two proximal screws and two distal screws were inserted bicortically. In addition, a 4.0-mm cannulated crossed screw of the same manufacturer was used. The screw was inserted from the dorsal and distal side of the first metatarsal bone in direction to the plantar and proximal side of the cuneiform bone. The plate was fixed at the plantar side (Fig. 2a).

*Double bridge plate® (Königsee Implantate GmbH, Allendorf, Germany)*: a titanium H-shaped locking plate with four holes for 2.7-mm angle-stable cortical screws, 22 mm long. Two proximal screws and two distal screws were inserted bicortically. In addition, a 4.0-mm cannulated crossed screw of the same manufacturer was used. The screw was inserted from the dorsal and distal side of the first metatarsal bone in direction to the plantar and proximal side of the cuneiform bone. The plate was fixed at the plantar side (Fig. 2b).

*IOFix® (Extremity Medicals, Parsippany, USA)*: an angle-stable intramedullary fixation device with a two-part construct of a 5.0-mm-thick and 40-mm-long lag screw and a 8.0-mm-thick and 25-mm-long X-Post®. The X-Post® was implanted into the first metatarsal bone from the dorsal to the plantar side. Implantation was bicortically with a 1-cm bone bridge to the arthrodesis side. The lag screw was applied in a 60° angle into the cuneiform bone through the plantar cortex (Fig. 2c).

### Testing protocols

The following protocols were performed: a measurement of the initial compression forces in the arthrodesis gap (I), a non-destructive sinusoidal load test (II), and a load-to-failure test (III). In each protocol, force was applied directing from the plantar to the dorsal surface in a 90° angle to the ground to simulate standing and walking. The sinusoidal load test was used to simulate a postoperative patient, partially weight bearing in a healing shoe. After a preload of 10 N, cyclic loads between 5 and 50 N were applied sinusoidally with a frequency of 0.5 Hz and 5000 cycles. Then, stiffness of the fusion at the initial phase and at the end phase was compared by measurements of displacement of the bones with a laser. After the continuous load test, the load-to-failure test was performed. All of the three synthetic bones, each one of them with a different implant for osteosynthesis (see above), were loaded until fracturing.

### Statistics

Statistical analysis was performed with statistical software package SPSS® Version 24 (IBM, Armonk, North Castle, New York, USA). Compression forces were compared using one-way analysis of variance (ANOVA) followed by post hoc tests with Bonferroni correction for multiple testing. The non-destructive loadings were compared using ANOVA with repeated measures followed by post hoc tests with Bonferroni correction. The repeated measures are an initial test of displacement, a stress phase, and the final test of displacement. For the load-to-failure test, Kaplan-Meier curves were generated from survival data of the implants and groups were compared using log-rank test, the Tarone-Ware test, and the modified Wilcoxon test. A *P* value of less than 0.05 was considered significant.

## Results

### Test protocol I

**Fig. 3:**
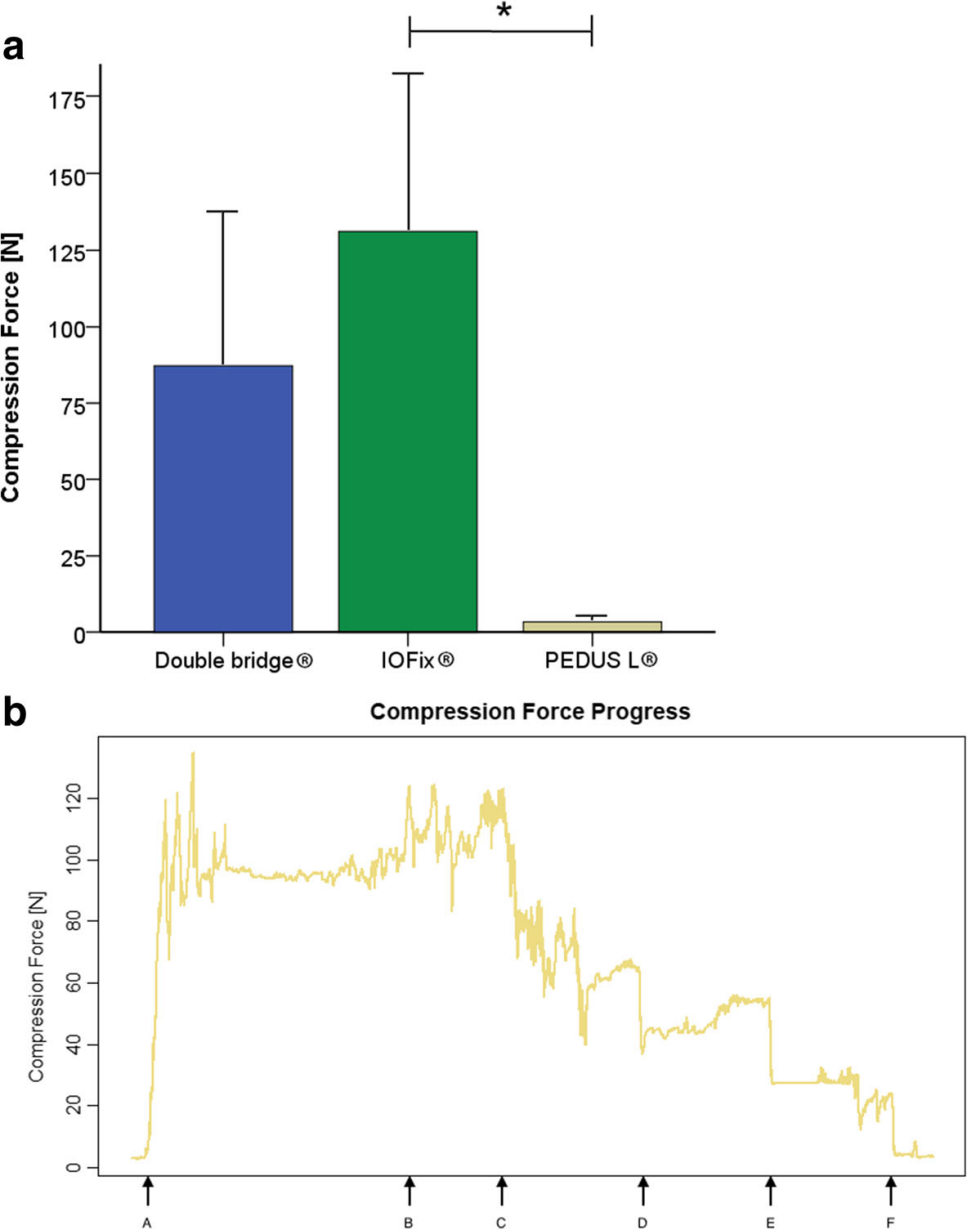
**a** Initial compression forces measured directly after osteosynthesis in newton. Differences between the groups were significant over all (*P = 0.033, corrected by Bonferroni). **b** Compression force progress for the plantar locking plate. Points of the steps of osteosynthesis were documented at the *x*-axis

Compression of bone fragments is a known promoter of consolidation. Therefore, compression forces between the different synthetic bones were measured after the osteosynthesis was performed. The highest initial compression force was provided by the IOFix® implant (131 ± 55 N), followed by the medial locking plate (87 ± 51 N) and the plantar plate (3 ± 1 N,) (Fig. 3a). The low initial compression force values for the plantar plate construct where measured as recently as the second locking screw was applied beyond the arthrodesis gap (Fig. 3b). The initial compression force provided by IOFix® implant differed from the force provided by the PEDUS-L® (*P* = 0.033). Taken together, these data suggest that the IOFix® device provided the highest compression force.

### Test protocol II

**Fig. 4:**
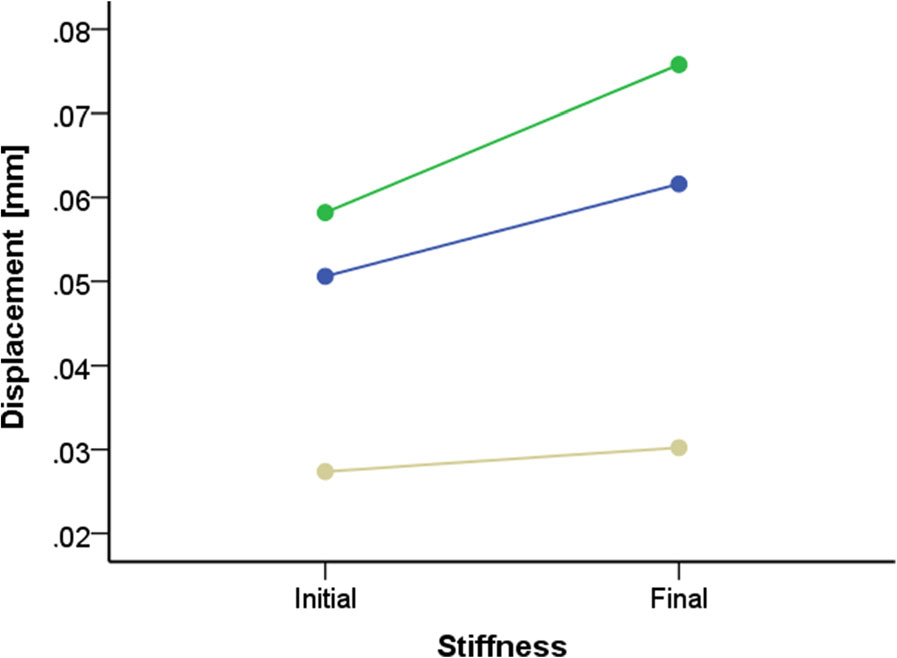
Displacement [mm] of the bones at the fusion side. Test included a comparison of the initial stiffness and the stiffness after the cyclic test procedure

The second test protocol was performed to analyse the stability of the osteosynthesis while simulating weight-bearing in a healing shoe. Differences between the initial and the final displacement of the synthetic bones and differences between the three bone-implant-combinations were analysed (Fig. 4): while the IOFix® combination and the Double bridge plate® combination showed significant loosening after the 5000 cycles, the PEDUS L Plantar Lapidus Plate® combination demonstrated rigid conditions after the test (Table 1). The stiffness provided by the IOFix® combination did not differ compared to the stiffness provided by the Double bridge plate® combination (*P* = 0.143). Furthermore, the stiffness provided by the PEDUS L Plantar Lapidus Plate® combination was superior compared to both of the other fixation methods (vs. Double bridge plate® *P* ≤ =0.000, vs. IOFix® *P* ≤ 0.000). Taken together, fixation with the plantar plate combination demonstrated a higher stiffness after the non-destructive sinusoidal load test compared to fixation with the IOFix® implant or the medial locking plate.

**Table 1:** Displacement between the cuneiform and the metatarsal bones before and after performing the test protocol II in millimetre

	IOFix® mean (SD)	Double bridge plate® mean (SD)	Pedus L Plate® mean (SD)
Initial displacement	0.058 ± 0.005	0.051 ± 0.003	0.027 ± 0.005
Final displacement	0.076 ± 0.017	0.062 ± 0.002	0.030 ± 0.007

### Test protocol III

**Fig. 5:**
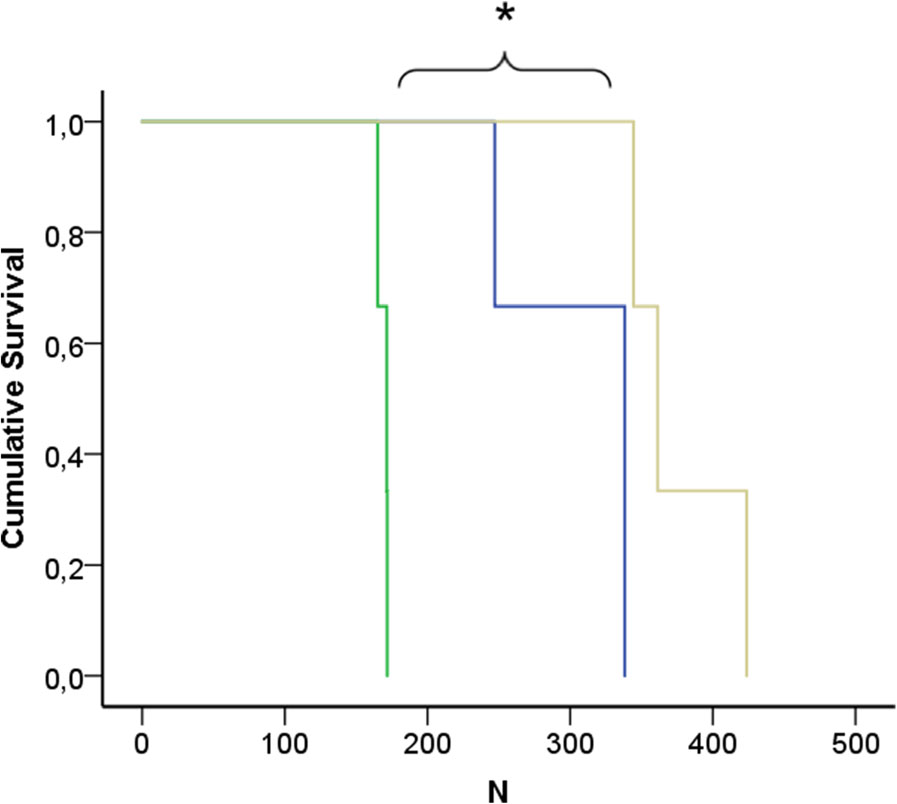
Cumulative survival of the three implant model groups related to the force due to failure in newton. The intramedullary implant showed the earliest failure, followed by the medial locking plate and the plantar locking plate (*log-rank test *P* = 0.002, Tarone-Ware test *P* = 0.005, and the modified Wilcoxon test *P* = 0.003)

The load-to-failure test was performed for all nine bone-implant-combination models to test the stability of the different implant devices until fracture of the bone. Failure was recognised in the following ranking: (1) the angle-stable intramedullary IOFix® fixation device (173 ± 8 N), (2) the medial located Double bridge plate® (324 ± 24 N), and (3) the PEDUS L Plantar Lapidus Plate® (377 ± 41 N, Fig. 5).

## Discussion

The aim of this study was to compare actual implants for the fusion of the first tarsometatarsal joint in hallux surgery. Initial compression force and stiffness during postoperative weight-bearing was examined. The intramedullary device (IOFix®) demonstrated the highest initial compression force among the three tested implants. The plantar locking plate showed the best overall stability during cyclic weight-bearing simulation and had the lowest interfragmentary diastase.

Many study groups use cadaver specimens for implant research [12, 15, 16]. Cadaver specimens offer conditions similar to an in vivo setting (ligaments, periosteum, bone mineral density). In contrast, synthetic bones offer identical bone shapes and densities for all study samples and therefore provide an exact and reproducible experimental setting [17, 18].

The basic experimental set-up of this study was chosen based on the work of Roth and coworkers [12]. While Roth and colleagues used a distance-controlled method of force application and measurements, the present study was performed in a frequency-controlled set-up with a value recalculation by the intercept theorem. Using the latter method, measurements of the fusion gap between the two synthetic bones become more accurate because side-effects like plastic strains of the bone or of the fixation in the mounting device are eliminated.

Comparisons of different fixation devices for the modified Lapidus procedure were also performed in previous studies [12, 14–16]. Knutsen and colleagues compared fixations with crossed screws, a dorsal locking plate without a crossed screw, and the IOFix® device with each other [14]. They found a higher failure load for the IOFix® compared to the crossed screw procedure and the dorsal locking plate fixation. In contrast to the approach reported by Knutsen and colleagues, in the current study, a compression screw was inserted in every implant group to evaluate the initial compression force. Roth and colleagues found a greater stability of the fixated bone using a plantar locking plate compared to the IOFix® [12]. Furthermore, Klos and colleagues confirmed an advantage of a plantar orientation of implants when they compared fixation with a medial locking plate with crossed screws and in another study a plantar against a dorsomedial locking plate [15, 16]. Similar to those findings, in the current study, the plantar locking plate showed the highest stability (Fig. 4) and the highest failure load (Fig. 5).

Interestingly, there was a complete loss of compression after the locking plate was applied. The high rigidity of the PEDUS L® Plate, which is able to override the initial compression by the crossed screw, might explain this effect. However, this compression loss was not seen when the Double bridge plate® was used. Because the thicker plate design could be responsible for this finding, further research should address which types of locking plates are able to override the compression of a screw. These devices might be sufficient to fix the bone fragments with a forceps rendering a screw unnecessary. The intramedullary IOFix® device showed the earliest failure and the lowest stability during test protocol II. Therefore, advantages like a small incision and fast technique have to be balanced against outcome-relevant parameters such as failure rate and stability. Further research should be performed to investigate if a longer postoperative immobilisation or the use of an additional, second intramedullary device could improve implant stability. In summary, choosing the right implant for arthrodesis of the first tarsometatarsal joint has to remain an individual decision for every surgical case. Whether the use a rigid plantar plate with high stability is associated with a decreased non-union rate should be addressed in future research.

## Conclusion

The modified Lapidus procedure is a common treatment of medial deviation of the first metatarsal bone in hallux valgus disease. Modern fixation devices provide a higher stability and earlier weight-bearing than crossed screws. The intramedullary device (IOFix®) demonstrated the highest initial compression force of the three tested implants. The plantar locking plate showed the best overall stability during cyclic weight-bearing simulation and had the lowest interfragmentary diastase. Further research with clinical data is necessary to study if the IOFix® needs a longer period of non-weight-bearing to reach a better non-union rate compared to locking plates and if compression screws are unnecessary in addition to locking plates.

## Abbreviation

SD: Standard deviation

## References

[1] Durman DC (1957). Metatarsus primus varus and hallux valgus. AMA Arch Surg.

[2] Hueter C (1870). Klinik der Gelenkkrankheiten mit Einschluss der Orthopädie.

[3] Fraissler L, Konrads C, Hoberg M, Rudert M, Walcher M (2016). Treatment of hallux valgus deformity. EFORT Open Rev.

[4] Sharma J, Aydogan U (2015). Algorithm for severe hallux valgus associated with metatarsus adductus. Foot Ankle Int..

[5] Cottom JM, Vora AM (2013). Fixation of lapidus arthrodesis with a plantar interfragmentary screw and medial locking plate: a report of 88 cases. J Foot Ankle Surg.

[6] Klaue K, Hansen ST, Masquelet AC (1994). Clinical, quantitative assessment of first tarsometatarsal mobility in the sagittal plane and its relation to hallux valgus deformity. Foot Ankle Int..

[7] Baravarian B, Briskin GB, Burns P (2004). Lapidus bunionectomy: arthrodesis of the first metatarsocunieform joint. Clin Podiatr Med Surg.

[8] Lapidus PW (1934). Operative correction of the metatarsus primus varus in hallux valgus. Surg Gynecol Ostet.

[9] Easley ME, Trnka HJ (2007). Current concepts review: hallux valgus part II: operative treatment. Foot Ankle Int.

[10] Coughlin MJ (1996). Hallux valgus. J Bone Joint Surg Am.

[11] Coughlin MJ, Mann RA, Coughlin MJ, Mann RA, Saltzman CL (2007). Hallux valgus.

[12] Roth KE, Peters J, Schmidtmann I, Maus U, Stephan D, Augat P (2014). Intraosseous fixation compared to plantar plate fixation for first metatarsocuneiform arthrodesis: a cadaveric biomechanical analysis. Foot Ankle Int..

[13] Perren SM (1979). Physical and biological aspects of fracture healing with special reference to internal fixation. Clin Orthop Relat Res.

[14] Knutsen AR, Fleming JF, Ebramzadeh E, Ho NC, Warganich T, Harris TG, Sangiorgio SN (2017). Biomechanical comparison of fixation devices for first metatarsocuneiform joint arthrodesis. Foot Ankle Spec.

[15] Klos K, Gueorguiev B, Mückley T, Fröber R, Hofmann GO, Schwieger K, Windolf M (2010). Stability of medial locking plate and compression screw versus two crossed screws for lapidus arthrodesis. Foot Ankle Int..

[16] Klos K, Simons P, Hajduk AS, Hoffmeier KL, Gras F, Fröber R, Hofmann GO, Mückley T (2011). Plantar versus dorsomedial locked plating for Lapidus arthrodesis: a biomechanical comparison. Foot Ankle Int..

[17] Cristofolini L, Viceconti M (2000). Mechanical validation of whole bone composite tibia models. J Biomech.

[18] Heiner AD, Brown TD (2001). Structural properties of a new design of composite replicate femurs and tibias. J Biomech.

